# Rating scales for cervical dystonia: a critical evaluation of tools for outcome assessment of botulinum toxin therapy

**DOI:** 10.1007/s00702-012-0887-7

**Published:** 2012-08-17

**Authors:** Wolfgang H. Jost, Harald Hefter, Andrea Stenner, Gerhard Reichel

**Affiliations:** 1 Department of Neurology, Deutsche Klinik für Diagnostik, Wiesbaden, Germany; 2 Department of Neurology, University of Düsseldorf, Düsseldorf, Germany; 3 Department of Movement Disorders, The Paracelsus Clinic, Zwickau, Germany

**Keywords:** Cervical dystonia, Rating scales, Botulinum toxin, Outcome assessment

## Abstract

Botulinum neurotoxin is the therapy of choice for all forms of cervical dystonia (CD), but treatment regimens still vary considerably. The interpretation of treatment outcome is mainly based on the clinical experience and on the scientific value of the rating scales applied. The aim of this review is to describe the historical development of rating scales for the assessment of CD and to provide an appraisal of their advantages and drawbacks. The Tsui score and the Toronto Western Spasmodic Torticollis Rating Scale (TWSTRS) have been widely employed in numerous clinical studies as specific instruments for CD. The obvious advantage of the Tsui score is its simplicity so that it can be easily implemented in clinical routine. The TWSTRS allows a more sophisticated assessment of functional features of CD, but only the Tsui score includes a rating for tremor. Other benefits of the TWSTRS are the disability and pain subscales, but despite its value in clinical trials, it might be too complex for routine clinical practice. None of the rating scales used at present has been rigorously tested for responsiveness to detect significant changes in clinical status after therapeutic interventions. Moreover, clinical data support a new classification of CD leading to a differentiation between head and neck subtypes. As the current rating scales are not able to cover all these aspects of the disorder, further research is needed to develop a valid and reliable instrument which considers the most current classification of CD.

## Introduction

Idiopathic cervical dystonia (CD) is the most frequent form of focal dystonia encountered in neurological practice. CD is characterized by involuntary contractions of specific muscles leading to abnormal movements of the head and/or unintentional adoption of sustained and frequently painful postures of the head, neck and shoulders. Botulinum neurotoxin (BoNT) has become the established treatment of choice, and the American Academy of Neurology (AAN) issued a level A recommendation (Simpson et al. 2008). About 80 short-term and long-term studies in CD patients have been published (Kamm and Benecke 2011; Costa et al. 2005a, b, c; Truong and Jost 2006) applying a variety of objective and subjective rating scales to evaluate the outcome of treatment with BoNT.

Differential diagnosis and treatment of CD are mainly based on the clinical assessment. Diagnostic aids such as computer tomographic scans or magnetic resonance imaging can provide some additional information in specific cases. However, in routine practice, determination of different subtypes of CD and BoNT injection of the affected muscles depends on the diagnostic skills of the treating physician. Furthermore, there are no objective functional parameters for CD such as blood pressure in hypertension or lung function in asthma patients which can be precisely measured using manual or electronic devices. This emphasizes the need for reliable rating scales that cover the most important aspects of different subtypes of CD.

An ideal rating scale should be as short and simple as possible in order to be implemented across multicenter clinical studies as well as in daily clinical practice. Each item of the scale has to be clearly defined to guarantee a high inter-rater reliability. Applying a rating scale, the assessor should be able to discriminate between subtypes of CD and to quantify the effect after administration of treatment. In addition, rating scale for CD should cover those aspects which are most important to patients. Not only functional disability but also the influence on activities of daily living and the psychosocial burden imposed on patient’s well-being need to be considered.

Precise tools to rate improvement or deterioration are important for any disorder to assess the patient’s disease state as well as the outcome after treatment. The aim of this review is to describe historical and current rating scales used to evaluate CD and to provide a critical assessment taking into account the classification of different CD subtypes and dystonic tremor.

## Rating scales for CD

Several different rating scales have been proposed to evaluate CD. The Fahn–Marsden dystonia scale has been developed for the assessment of generalized dystonia, originally for use in a therapeutic trial of trihexyphenidyl (Burke et al. 1985). The scale rates the severity of movements affecting different body parts, each on a 5-point scale, but it includes only one item of CD (i.e. neck). The Fahn–Marsden scale also takes into account provoking factors: 1 (dystonia appearing only with action) and 4 (persistent dystonia at rest). Truncal and limb movements are assigned a weight of 0.5 and cranial movements are assigned a weight of 1.0 for a maximal total score of 120. In addition, there is a separate disability scale for activities of daily life (speech, handwriting, feeding, eating/swallowing, hygiene, dressing, walking) rated from 1 (normal) to 4 (complete disability).

In 1997, a consensus conference of dystonia experts developed the Unified Dystonia Rating Scale (UDRS) as a simple tool to evaluate the severity of different forms of focal dystonia (Comella et al. 2003). The UDRS includes two components, a duration factor and a motor severity factor for 14 regions of the body. The duration factor is a 9-point scale with 0.5 steps from 0 to 4. Similar to the Fahn–Marsden dystonia scale, only one item of the UDRS severity scale applies to CD (i.e. neck). Severity of movements is rated on the following 5-point scale: 0 = none, 1 = mild (≤25 % of possible normal range), 2 = moderate (>25 % but ≤50 % of possible normal range), 3 = severe (>50 % but ≤75 % of possible normal range), 4 = extreme (>75 % of possible normal range). Although applicable for generalized dystonia, neither the Fahn–Marsden scale nor the UDRS is considered specific for CD.

Fahn (1989) worked on a rating scale particularly designed for the assessment of CD resulting in the Columbia Torticollis Rating Scale. This composite scale consists of ratings for head movements (torticollis rating) and disability scales. The torticollis scale documents the direction of movement, the circumstances when the head deviation is present, the duration of deviation while sitting, the range of active movement, the excursion amplitude, the duration and severity of pain while sitting, the degree of reduction in deviation with the use of sensory tricks, the average frequency, duration and severity of forceful spasms, the presence of tremor and gross jerking movements of the head, and the presence of essential tremor of the hands. The disability scale assesses the limitation of functional activities such as driving, reading, watching television, going out to movies, shopping, walking about, feeding, falling asleep, and performance of housework or outside work. The value of this detailed scale for descriptive clinical assessment was demonstrated in a study defining the clinical characteristics of a large cohort of CD patients (Chan et al. 1991). However, the complexity might have prevented broad use in clinical practice, and the utility for clinical trials remains unclear as it was only applied in one double-blind, controlled trial (Table 1, Greene et al. 1990).

**Table 1:** Use of rating scales in pivotal clinical studies with BoNT treatment for cervical dystonia (studies listed in chronological order)

References	Study design	BoNT	Number of CD patients	Efficacy outcome measure(s)	Results/remarks
*Placebo-controlled studies*					
Greene et al. (1990)	Randomized, double-blind, placebo-controlled, parallel groups	BoNT-A (Botox^®^)	55	GIR (+3 = markedly improved, −2 = definitely worse); VAS for functional capacity and for pain; degree of head turning; Columbia Torticollis Rating Scale	BoNT-A significantly improved the severity of CD, disability, pain and degree of head turning
Brans et al. (1996)	Randomized, double-blind, double-dummy, comparator-controlled, parallel groups	BoNT-A (Dysport^®^)	66	TWSTRS-Disability; TWSTRS-Pain; Tsui score; HRQoL scale	TWSTRS-Disability (primary outcome), Tsui score and HRQoL scale were significantly in favor of BoNT-A compared to trihexiphenidyl
Lew et al. (1997)	Randomized, double-blind, placebo-controlled, parallel groups	BoNT-B (Myobloc™/Neurobloc^®^)	122	TWSTRS-Total score; subscores for severity, disability and pain; VAS for pain; VAS for investigator and patient global assessment of change; Sickness Impact Profile scores	TWSTRS-Total score (primary outcome) improved for all treatment groups (including placebo), but BoNT-B was significantly superior. Improvement with BoNT increased with higher doses
Poewe et al. (1998)	Randomized, double-blind, placebo-controlled, parallel groups	BoNT-A (Dysport^®^)	75	Tsui score (modified version); pain on 4-point scale; global assessment of improvement post injection; global rating of efficacy; need for retreatment at week 8	Magnitude of improvement was greatest after 1,000 U BoNT-A, but with significantly more AEs; lower start dose of 500 U BoNT-A is recommended
Brin et al. (1999)	Randomized, double-blind, placebo-controlled, parallel groups	BoNT-B (Myobloc™/Neurobloc^®^)	77	TWSTRS-Total score; subscores for severity, disability and pain; VAS for pain; VAS for investigator and patient global assessment of change	Significant difference in favor of BoNT-B for primary (TWSTRS-Total score) and all secondary outcome variables
Brashear et al. (1999)	Randomized, double-blind, placebo-controlled, parallel groups	BoNT-B (Myobloc™/Neurobloc^®^)	109	TWSTRS-Total score; subscores for severity, disability and pain; VAS for pain; investigator and patient global assessment of change	The mean improvement in TWSTRS-Total score (primary outcome) was significantly in favor of BoNT-B, but higher for the 10.000 U compared to the 5.000 U dose
Truong et al. (2005)	Randomized, double-blind, placebo-controlled, parallel groups	BoNT-A (Dysport^®^)	80	TWSTRS-Total score; subscores for severity, disability and pain; VAS for pain	Improvement in TWSTRS-Total score was the result of improvement in each of the three subscale scores. BoNT-A improved not only head position but also pain and disability
*Comparator-controlled studies*					
Benecke et al. (2005)	Randomized, double-blind, comparator-controlled, parallel groups	Xeomin^®^ versus Botox^®^	463	TWSTRS-Severity; TWSTRS-Pain; VAS for pain; 9-point Global Response Scale; responder rates; investigator global assessment of efficacy	Improvement in TWSTRS-Severity score (primary outcome) 4 weeks after Xeomin^®^ was non-inferior to Botox^®^
Pappert and Germanson (2008)	Randomized, double-blind, comparator-controlled, parallel groups	BoNT-A vs. BoNT-B	111	TWSTRS-Total score; subscores for severity, disability and pain; VAS for pain; investigator and patient global assessment	Improvement in TWSTRS-Total score (primary outcome) 4 weeks after BoNT-B was non-inferior to BoNT-A

*AEs* adverse events; *BoNT* botulinum neurotoxin; *GIR* Global Improvement Rating; *HRQoL* health-related quality of life; *TWSTRS* Toronto Western Spasmodic Torticollis Rating Scale; *U* units; *VAS* Visual Analogue Scale

Also in the 1980s, a comparably brief rating scale for CD was developed by Tsui et al. (1985, 1986, 1987) and Stell et al.  1988). It is an impairment scale which evaluates the amplitude and duration of sustained posture and intermittent movements of the head, as well as the presence of shoulder elevation and tremor. Rotation, tilt, and sagittal movements are rated on a 0–3 scale for a maximum of 9. Additionally, head tremor is rated from 0 to 2 and shoulder elevation from 0 to 3. Multiplication by a duration score is performed for amplitude of sustained movements (1 = intermittent, 2 = constant) and for tremor (1 = occasional, 2 = continuous) resulting in a total possible score of 25. Poewe et al. (1998) suggested a modification of the Tsui score to increase the sensitivity of the scale for postural head deviations. Four different modes of postural activation were performed to assess the amplitude of sustained movements (sitting, lying, standing, walking).

Another brief scale has been described by the research group of Lang et al. specifically for the assessment of CD (Weiner and Lang 1989). The degrees of turn (chin to side of turn) plus degrees of tilt (ear down toward shoulder) are rated in steps of 15° on a scale from 0 to 6, and sagittal movements (anterocollis or retrocollis) are rated from 0 to 3. These are added for a maximal score of 15 and multiplied by a severity factor (0 = none to 4 = severe) leading to a maximum sum score of 60. In addition, the time to a maximum of 60 s that the patient is able to hold the head fixed in the central position is also measured. This scale was subsequently altered and expanded to form the Toronto Western Spasmodic Torticollis Rating Scale (TWSTRS) Severity scale described below.

The TWSTRS is a composite scale which covers different features of CD (Consky et al. 1990; Consky and Lang 1994; Comella et al. 1992; Dubinsky et al. 1991). The first part is based on the physical findings (severity subscale), the second part rates disability, and the third part pain. Details of the TWSTRS are displayed on the Web site http://www.wemove.org. The TWSTRS-Severity scale includes the following items: A. maximal excursion (rotation, tilt, anterocollis or retrocollis, lateral shift, sagittal shift), B. duration factor, C. effect of sensory tricks, D. shoulder elevation/anterior displacement, E. range of motion (without the aid of sensory tricks), F. time (up to 60 s that the patient is able to maintain the head within 10° of the neutral position without the use of sensory tricks). The sum of A to F amounts to a maximum score of 35 with the duration factor weighted twice.

The TWSTRS-Disability is a six-item scale that comprises an assessment of performances of daily activities which may be possibly affected by CD: work performance (job or domestic), activities of daily living (feeding, dressing, hygiene), driving, reading, watching television, and leisure activities outside the home. Each item is rated on a 6-point scale (0 = no difficulty, 5 = highest degree of disability). The TWSTRS-Pain consists of a severity score for the patient’s usual, worst, and best pain in the last week, as well as a duration component and an assessment of the contribution of pain to disability. The score range is between 0 and 20, with 20 assigned to the highest possible experienced pain.

In addition to rating scales assessing the functional impairment due to CD, some research groups have made attempts to implement instruments for quantification of health-related quality of life (HRQoL) (Cano et al. 2004b; Slawek et al. 2007). HRQoL instruments should on the one hand be relevant to patients and on the other hand sensitive enough to assess functional health and treatment effects in clinical trials and in daily practice. The aim was to develop a disease-specific questionnaire that addresses the perceptions and concerns of patients with CD. Cano et al. (2004a, 2006) created a 58-item rating scale (CD Impact Profile, CDIP-58) measuring the health impact of CD in eight areas (head and neck symptoms, pain and discomfort, activities, walking, sleep, annoyance, mood, and psychosocial functioning). The research of the Austrian Botulinum Toxin and Dystonia Study Group (Müller et al. 2004) resulted in a shorter, 24-item questionnaire (CDQ-24) based on 5 subscales: stigma, emotional well-being, pain, activities of daily living, and social/family life. Each item is scored from 0 (never) to 4 (always), representing increasing severity of impairment. HRQoL questionnaires can be used in combination with clinical rating scales of dystonia severity to capture the status of patient’s well-being.

## Validation of rating scales for CD

The Fahn–Marsden rating scale for generalized dystonia has been validated by assessment of videotapes (Burke et al. 1985). In this evaluation, 10 patients were rated on a simple global scale from 0 to 5 and in parallel by the Fahn–Marsden rating scale. All patients were rated twice by two assessors. A close correlation (*r* = 0.9) was found between different raters and an almost 100 % correlation between repeated ratings, demonstrating good inter-rater and intra-rater reliabilities. In a validation study with 25 dystonia experts, the UDRS showed a very good correlation with the Fahn–Marsden dystonia scale. Inter-rater agreement was fair to excellent with intraclass correlation coefficients ranging from 0.71 to 0.78 (Comella et al. 2003). The modifying ratings (duration of the UDRS and provoking factor in the Fahn–Marsden scale) showed less agreement than the motor severity ratings. Neither the Fahn–Marsden scale nor the UDRS have been tested for clinical responsiveness to therapeutic interventions or reproducibility (Jankovic and Tolosa 2007).

There are a couple of publications about the reliability of rating scales specific for CD. The inter-observer variability of the Tsui score was determined by means of randomized videotape recordings which were again scored “blind” by another physician (Tsui et al. 1986). The inter-observer correlation (0.86–0.87) showed that the scale gives reproducible results (Tarsy 1997). However, scatter between rater scores for any individual patient has been high (Gelb et al. 1989; Moore and Blumhardt 1991). Furthermore, in some studies, there have been major differences between scores and patient’s subjective assessments of therapeutic response (Gelb et al. 1989; Blackie and Lees 1990). The Cervical Dystonia Severity Scale, which is very similar to the Tsui score, was evaluated in 42 patients with CD rated by two different assessors at each of four participating centers twice in the same day. The scale was very reproducible within and between different raters, with correlations ranging from 0.79 to 0.94 (O’Brien et al. 2001).

The TWSTRS is a validated scale which has been frequently applied in clinical trials as the primary outcome parameter. A total of 200 CD patients were videotaped by using the TWSTRS videotape protocol and independently assessed by three movement disorder specialists. The panel rated the videotape segments of the individual subsections of the TWSTRS and subsequently, the full patient cases (Comella et al. 1997). The rates of agreement for all individual components of the TWSTRS and the total TWSTRS were statistically significant (all *p* < 0.01). The inter-rater agreement was highest for rotation, anterocollis, and retrocollis and lowest for lateral shift. In addition, a substantial correlation could be demonstrated for the change in TWSTRS-Severity score after BoNT treatment and patient perception of overall improvement as well as for the change in disability and pain scores (Consky et al. 1990; Consky and Lang 1994).

The validity and reliability of the disease-specific HRQoL questionnaire CDQ-24 were determined in 231 patients with cranial and cervical dystonia. The evaluation also included the Short Form-36 Health Survey (SF-36) as a general HRQoL instrument, the Tsui score to assess severity of CD, and a global assessment of pain (Müller et al. 2004). High correlations were found between those CDQ-24 and SF-36 subscales that measure similar aspects. Patients with CD showed low correlations of the CDQ-24 subscores with the Tsui score, which could be expected as the Tsui score rates functional impairment and does not include HRQoL. However, correlations of CDQ-24 subscores with pain ratings were higher. The CDQ-24 questionnaire showed good reliability properties and appears to be sensitive to changes that are important to patients.

## Use of rating scales for CD in clinical studies with botulinum toxin

Of the approximately 80 studies with BoNT in CD which have been published, 14 are controlled studies (Kamm and Benecke 2011). Of these seven randomized, double-blind studies were classified as class I evidence by the AAN, four studies with BoNT-A and three studies with BoNT-B (Simpson et al. 2008). For the tabulated overview (Table 1), another double-blind class I study comparing BoNT-A and BoNT-B (Pappert and Germanson 2008; Kamm and Benecke 2011) and the comparator-controlled study of Benecke et al. 2005 (class II), which was pivotal for the marketing authorization of Xeomin^®^, were incorporated.

Five of the pivotal studies compiled in Table 1 used the TWSTRS-Total score as the primary outcome parameter (Lew et al. 1997; Brin et al. 1999; Brashear et al. 1999; Truong et al. 2005; Pappert and Germanson 2008). Only in one of these studies, the Tsui score was applied as primary outcome (Poewe et al. 1998). Nevertheless, in the 1980s and 1990s, the Tsui score was implemented in several other controlled studies to assess the treatment effect of BoNT (Kamm and Benecke 2011). A couple of studies only applied a TWSTRS subscore as the primary efficacy parameter, e.g. the TWSTRS-Severity score in the study of Benecke et al. 2005. The TWSTRS-Disability was the primary outcome in the study of Brans et al. (1996), whereas TWSTRS-Pain and Tsui score were used, in addition, as secondary parameters. Greene et al. (1990) conducted the only study which employed the Columbia Torticollis Rating Scale. However, the report of the study does not present the outcome for the total score, but describes the response to BoNT injections primarily by a patient-based Global Improvement Rating (GIR) from +3 (markedly improved) to −2 (definitely worse).

There are a few studies applying several different rating scales in order to determine which is the most suitable to evaluate treatment effects of BoNT in patients with CD. Tarsy (1997) assessed 76 consecutive idiopathic CD patients with both Tsui score and TWSTRS before and after injection of BoNT. Tsui and TWSTRS-Total score reduction rates after treatment correlated significantly (Pearson correlation coefficients 0.57; <0.0001). Also Tsui and TWSTRS-Severity, which are both objective ratings of clinical severity, showed a comparable high correlation. In contrast, there was only weak or no correlation of TWSTRS-Pain score reduction with either of the objective severity scales. Based on these results, the author suggests that an objective scale of severity such as either the Tsui score or the TWSTRS severity subscale in conjunction with the TWSTRS pain subscale adequately assesses the improvement of CD following treatment with BoNT. Odergren et al. (1994) come to a similar conclusion and recommend using a combination of Tsui score for dystonic posture and movement ability and a Visual Analogue Scale (VAS) for pain to assess the efficacy of BoNT. In accordance with the results of Tarsy (1997) described above, another study in 64 CD patients receiving BoNT injections demonstrated a poor correlation between motor findings from the Tsui score and pain and disability sections of the TWSTRS (Lindeboom et al. 1998). In this study, there were no differences between the effect sizes of impairment and pain of patients who continued treatment and drop-outs. This suggests that these outcome measures do not appropriately reflect BoNT efficacy. The authors conclude that disability, handicap and a global measure of disease burden were the most suitable outcome parameters to determine the clinical efficacy of BoNT.

## Assessment of rating scales for CD

The efficacy of BoNT in idiopathic CD has been evaluated in a huge number of clinical studies, but there is still no final clue which is the optimal rating scale to assess treatment effects. This section will provide a thorough appraisal of the advantages and drawbacks of Tsui score, TWSTRS, and UDRS, which are the most current rating scales used in clinical studies as well as in daily clinical practice.

The **UDRS** is a simple, validated rating scale for generalized assessment of focal dystonia and comprises duration as well as a severity factor. The duration rating of the UDRS parallels the duration factor of the TWSTRS. The severity of abnormal movements can be uniformly described in steps of 25 % for dystonia in different regions of the body. An obvious advantage of the UDRS is that the scale can be easily implemented across multicenter studies with many investigators and in daily practice to evaluate patients with different forms of focal dystonia.

The main drawback for the assessment of CD is that the UDRS includes only one item for CD (i.e. neck) and is, therefore, not precise enough to describe disease-specific features. Severity is rated from 0 to 4 in steps of 25 %, but there is no definition which degree of excursion corresponds to each of the numerical ratings. Furthermore, the UDRS does not include ratings for pain and disability, i.e. factors that are important to patients. The effect of sensory tricks is not covered, and the scale does not take into account if the patient is able to change the dystonic posture. Altogether, the UDRS is not an adequate tool to assess the complex clinical picture of CD neither in daily practice nor in clinical trials.

The scoring system developed by Tsui comprises a rating for sustained movement amplitudes, duration, shoulder elevation and, in addition, for dystonic tremor. The Tsui score is a brief and relatively simple rating scale specific for CD which can easily be implemented in daily clinical routine. However, due to its simplicity, several features of CD regarded as relevant by patients are not covered, e.g. the scale does not include a rating for pain, disability, and HRQoL. In consequence, it shows only low correlation with HRQoL scales, which might be the explanation that in some studies there have been major differences between Tsui score and patient’s subjective assessments of therapeutic response. A deficiency for the use in multicenter studies by different investigators is the lack of a precise definition under which conditions the assessment should be carried out (e.g. sitting, eyes closed etc.). Different modes of postural activation (sitting, lying, standing, walking) can thus lead to diverging results. For the sake of reliability, it is essential that all potential assessors have the same understanding of each single item of a rating scale. The Tsui score uses the designations “mild”, “moderate”, and “severe” for some ordinal scores (e.g. for anterocollis and retrocollis) but a clear criterion definition for these ratings is missing.

Another immanent disadvantage of this scale lies in the fact that mixed forms of CD are generally assigned higher scores than simple forms, and thus, the sum score does not provide an appropriate measure of the extent of impairment. The Tsui score does not comprise a separate item for shift. In consequence, shift is underestimated in the total score as the degree of head deviation is minor compared to torticollis or laterocollis. Consky and Lang (1994) raised the point that measurements of sustained movement amplitudes are extremely difficult because maximal excursions may be sustained for only a short duration, and submaximal deviations may be otherwise constantly evident. Considering this fact, the simple dichotomous rating of 1 (intermittent) and 2 (constant) is not precise enough to describe the duration of sustained movements. It is also unclear if the evaluation of duration should only be based on the observation of the assessor or should also take into account the information provided by patients.

Further drawbacks of the Tsui score are that the scale does not include the effect of sensory tricks and that it captures only the abnormal posture but not the interaction of voluntary movements, whereas the TWSTRS includes a question if the patient is able to move the head toward or past midline. As an advantage compared to the TWSTRS, the Tsui score covers the assessment of dystonic tremor. But the rating of “continuous” for duration of tremor is questionable because the majority of the patients will not confirm that the tremor is present all the time without any interruption. The rating for tremor is also problematic from the statistical point of view. When multiplying tremor severity by duration, a result of 3 is not possible. The assumption of a Gaussian distribution cannot be made for statistical tests if values are missing within a score.

The **TWSTRS** covers the functional features of CD (severity subscale) as well as the aspects which are important to patients (disability and pain subscales). A positive correlation was demonstrated between change in severity score rated by physicians and patient’s self-reported improvement in disability and pain after treatment with BoNT (Consky et al. 1990). The TWSTRS is a validated scale including a videotape protocol such that patients are viewed in a standardized fashion. This ensures consistency and reproducibility across raters for multicenter trials. The TWSTRS videotape protocol is a valuable tool for a standardized assessment in clinical trials but might be too complex to be applicable in routine clinical practice. Similar to the Tsui score, the TWSTRS-Severity combines the amplitude of movements with a duration factor. In contrast to the dichotomous rating of duration (1 = intermittent, 2 = constant) of the Tsui score, the TWSTRS duration factor provides a more sophisticated assessment of duration on a 6-point scale in 25 % steps taking into account the proportion of time that the head deviation is most often maximal or submaximal in amplitude. Furthermore, the TWSTRS-Severity includes an item for the effect of sensory tricks.

Despite the obvious advantages of the TWSTRS, there are also some points of constructive criticism to be considered for the development of future scales. As already mentioned above, the TWSTRS does not enable to evaluate dystonic tremor; however, from the experience with the Tsui score, the difficulties to define a rating for tremor are evident. In addition, there is no scientific evidence to support the fact that rating of duration is weighted twice. The effect of tricks is rated from 0 (complete relief by one or more tricks) to 2 (little or no benefit from tricks). At bottom, a rating of 2 for the missing effect of tricks is not a measure of severity, and the fact that a patient who does not benefit from tricks does not have any impact on therapeutic decisions, but it is assigned the same weight, as for a moderate laterocollis. On the other hand, subtypes of CD which can be extremely disabling for the patient tend to be underestimated. Anterocollis can be assigned a maximum rating of 3—severe in case the chin approximates the chest because no other items (turn, tilt or shift) are applicable. Furthermore, items A. “Maximal excursion” and E. “Range of motion” of the TWSTRS-Severity scale basically describe the same aspects of abnormal posture, i.e. the ratings for these items are not independent from each other. Comella et al. (1997) raised some scale deficiencies already quite soon after the implementation of the TWSTRS. The author pointed out that there is no explicit definition of midline and full range for assessment of each of the three axes of movements.

Tsui score as well as TWSTRS has been used in numerous clinical studies and despite some drawbacks, as described above, proved their value to assess treatment effects of BoNT for decades. Although both rating scales are specific for CD, they are not able to cover all aspects of the disorder. Moreover, the phenomenological classification of CD has recently undergone a revision which challenges the accuracy of current rating scales. Are the rating scales for CD which are employed at present still valid to measure what they were originally designed to measure?

The goal of the investigation of Reichel (2011) in 78 patients with CD was to avoid primary non-responders to BoNT treatment and to inject those muscles that are causally involved in the pathology of the respective subtypes of dystonia. The author came to the conclusion that it is necessary to distinguish between neck and head types of CD (-collis and -caput) because different groups of muscles are affected. It was shown that in 20 % of the patients, the abnormal movement and/or posture only involves muscles which work on the atlanto-occipital joints (latero-, antero-, retro- or torticaput), and in further 20 %, it only affected muscles in the region of the cervical spine (latero-, antero-, retro- or torticollis). Sixty percent of the patients exhibited both head and neck types of CD but with a different degree of “caput” and “collis” involvement. Neither Tsui score nor TWSTRS-Severity allows a differentiation between -collis and -caput types of CD and, in consequence, need to be reworked to match the new phenomenological classification.

## Discussion and conclusions

Assessment of treatment outcome in clinical studies and in daily practice is only as reliable and valid as the instrument applied. Proper evaluation of patients with CD is a crucial point because clinical decisions and results of clinical studies are mainly dependent on the scientific quality of the rating scales employed. Although technical aids have been developed such as Zerviton^®^, a helmet system with custom-built software to measure dystonic postures of the head, these are not established in routine practice (Sommer et al. 2009). Treatment protocols for BoNT still differ considerably with regard to dose, injected muscles and number of injection sites. Progress in defining the most effective treatment approach and in ensuring comparability of results across several clinical studies significantly depends on the consensus about an appropriate rating scale which can be accepted as a standard throughout the scientific community.

Regardless of the rating scale applied, there are some inherent difficulties of measuring the severity of CD due to its variability depending on emotional stress, fatigue or activity. A rating scale for CD has to encompass the heterogeneity and variability of the clinical features of the disorder. It should include not only an assessment of the extent of dystonic movements but also for pain, disability, and quality of life, i.e. aspects which are most relevant to patients. None of the current rating scales reviewed above provides the possibility to rate all these complex aspects of the disorder.

The Scientific Advisory Group of the Medical Outcomes Trust (SAC 2002) recommended criteria for health measurement rating scales which represent the most current and complete guidelines. The SAC defined a set of eight key attributes for rating scales: conceptual and measurement model, reliability, validity, responsiveness, interpretability, respondent and administrative burden, alternate forms, and cultural and language adaptations. At a minimum, the evaluation of a rating scale should include reliability (the extent to which an instrument yields reproducible results), validity (the extent to which an instrument measures what it is designed to measure), and responsiveness (the extent to which a scale is responsive to detect a significant change in clinical status over time) (Consky and Lang 1994; Cano et al. 2004b). Furthermore, it is important that the content of a rating scale for CD is based on the actual conceptual disease model, e.g. the most current classification of CD, as well as empirically derived aspects from clinical practice. Finally, practical issues, such as the time taken to complete the scale, interpretability and considerations of patient burden are also relevant factors (SAC 2002; Cano et al. 2004b).

As illustrated in Table 2, the available CD-specific rating scales have not been developed strictly according to the guidelines described above. None of them cover at least the three essential criteria recommended by the Medical Outcomes Trust (reliability, validity, and responsiveness). Although Tsui score and TWSTRS have been widely used as primary outcome parameter in clinical studies, they have not been rigorously tested for responsiveness. Table 3 summarizes the pros and cons for Tsui score and TWSTRS which are the most frequently employed rating scales for CD in clinical practice as well as in clinical trials. An important drawback is that neither Tsui score nor TWSTRS reflects the most current conceptual model of CD. In a previous publication, Reichel (2011) describes a new classification for CD and points out the clinical importance to differentiate between head and neck subtypes. In consequence, there is an obvious deficiency to exactly describe all subtypes of CD with the rating scales available at present, and the need to develop a rating scale which includes the possibility to distinguish between “-collis” and “-caput” subtypes of CD.

**Table 2:** Evaluation of rating scales according to the Medical Outcomes Trust criteria

Rating scales	Criteria fulfilled		
	Reliability	Validity	Responsiveness
Fahn–Marsden scale	X	X	
Tsui score	X		
Toronto Western Spasmodic Torticollis Rating Scale (TWSTRS)	X	X	
Craniocervical dystonia questionnaire-24 (CDQ-24)	X		X

Modified from Cano et al. (2004b)

**Table 3:** Comparison of main features of commonly used rating scales for CD

Main features	Tsui score	TWSTRS
Brief and easy to apply	Yes	No
Standardized videotape protocol	No	Yes
Rating for amplitude of movements	Yes	Yes
Duration factor	Yes	Yes
Rating for shoulder elevation	Yes	Yes
Rating for shift	No	Yes
Rating for tremor	Yes	No
Effect of sensory tricks	No	Yes
Disability scale	No	Yes
Pain scale	No	Yes
Differentiation between -collis and -caput types of CD	No	No

As a first step to come to a consensus for an improved rating scale for CD, the authors collected their thoughts to the requirements for an “ideal” rating scale. A rating scale has to be easy to understand, easy to handle, and swiftly to apply. It must be reproducible if used by different raters and applicable in daily clinical routine as well as in clinical studies. Symptoms of the disorder should be rated by an ordinal numeric score, e.g. from 0 to 4 with increasing symptom severity. The aim of the scale is to cover all relevant symptoms of the target disease including those which are important to characterize the disorder but do not respond well to current treatment options. The same numeric scores should be used for each item of the rating scale. Particularly, the approach will be to assign the same weight to each item of the scale, although this might not be achievable for all items. At least, the attempt will be made that none of the symptoms are overrated or underestimated. Based on these assumptions, it should be possible to use the scores for statistical calculations.

The absolute sum score should provide a clear measure of the severity of the disease, the higher the value, the more the patient is affected. Accordingly, a reduction in score should closely correlate with therapeutic improvement. A rating scale has to be applicable for different therapeutic approaches and be sensitive enough to discriminate between less or more effective treatments. From the clinician’s perspective, it would be preferable that rating results could serve as guidance for selection of therapeutic algorithms and BoNT injection schemes. Furthermore, some components of the scale should be applicable to assess patient’s perspective of the disorder or even HQoL. Most likely, a rating scale for CD will comprise several subscales to cover symptom severity, impact on patient’s daily activities, and social burden of the disease.

Currently, we face the situation that none of the rating scales most frequently used in clinical practice offer the possibility to take a new classification of CD into account. A future rating scale should be designed to be independent of potential scientific findings which could change the diagnosis or classification of the target disease. These preliminary suggestions for the design of a standardized rating for CD represent only a first approach which needs to be further worked out with a panel of experts. During the development and validation process of the rating scale, it will turn out how many of the above-mentioned requirements can be put into practice or will remain wishful thinking.
